# Improving Ineffective Erythropoiesis in Thalassemia: A Hope on the Horizon

**DOI:** 10.7759/cureus.18502

**Published:** 2021-10-05

**Authors:** Ujjwal Madan, Himani Bhasin, Pooja Dewan, Jyotsna Madan

**Affiliations:** 1 Pediatrics, University College of Medical Sciences, Delhi, IND; 2 Pathology, Super Speciality Pediatric Hospital and Post Graduate Teaching Institute, Noida, Uttar Pradesh, IND

**Keywords:** jak-2 inhibitors, luspatercept, ruxolitinib, sotatercept, ferroportin inhibitors

## Abstract

Beta-thalassemia is an inherited hemoglobinopathy characterized by the impaired synthesis of beta-globin chains of hemoglobin leading to chronic hemolytic anemia. The mainstay of treatment for most patients remains regular blood transfusions and iron chelation. This conventional therapy has many limitations and challenges. Allogeneic hematopoietic stem cell transplant (HSCT) is the only available curative treatment but the availability of a suitable donor, financial constraints, and a need for specialist physicians can be limiting factors. Gene therapy is an upcoming curative therapeutic modality. An increased understanding of the underlying pathophysiology and molecular mechanisms of thalassemia has paved the way for novel pharmacological agents targeting ineffective erythropoiesis. These drugs act by decreasing transfusion requirements and hence decrease transfusion-related complications. The present review intends to provide an insight into the recent advances in pharmacological agents targeting ineffective erythropoiesis. Literature was searched and relevant articles evaluating newer drugs in thalassemia were collected from databases, including Pubmed, Scopus, Prospero, Clinicaltrials.gov, Google Scholar, and the Google search engine. We used the following keywords: thalassemia, novel, treatment, drugs, and ineffective erythropoiesis during the initial search. Relevant titles and abstracts were screened to choose relevant articles. Further, the full-text articles were retrieved and relevant cross-references were scanned to collect information for the present review.

## Introduction and background

Beta-thalassemia is an inherited hematological disorder caused by mutations in the beta-globin gene, which leads to insufficient beta-globin chain synthesis and, therefore, ineffective erythropoiesis [1]. The mainstay of treatment of beta-thalassemia includes life-long regular blood transfusions and iron chelation, resulting in severe complications compromising their overall survival [1]. Adverse effects associated with regular blood transfusions include systemic iron overload (cardiac, hepatic, endocrine, pulmonary dysfunction), infections, growth disturbances, and hemolytic and non-hemolytic blood transfusion reactions. Additionally, adverse effects of iron chelators used include gastrointestinal symptoms, arthralgia, skin rash, growth disturbances, visual defects, sensorineural hearing loss, and local infusion site reactions [1]. Although hematopoietic stem cell transplantation (HSCT) is curative, it is limited by the availability of human leukocyte antigen (HLA)-matched donors, the inherent risk of graft-versus-host disease (GVHD), and prohibitive costs. Recently, gene therapy using genetically modified autologous stem cells in thalassemia patients lacking a matched donor has rendered them transfusion-free, offering hope of cure [2].

Due to the limited availability of both these curative treatments, clinical studies have explored non-curative pharmacological treatment options such as erythropoiesis-stimulating agents that may prevent the complications associated with anemia, hemolysis, and iron overload [3-6]. Herein, we provide an insight into the recent advances in the drugs targeting ineffective erythropoiesis in beta-thalassemia. For the present review, we searched the literature and relevant articles evaluating newer drugs in thalassemia from databases, including PubMed, Scopus, Prospero, Clinicaltrials.gov, Google Scholar, and the Google search engine. The following keywords: thalassemia, novel, treatment, drugs, and ineffective erythropoiesis were used during the initial search. Relevant titles and abstracts were screened to choose relevant articles. Further, the full-text articles were retrieved and relevant cross-references were scanned to collect information for the present review.

## Review

Classification of newer pharmacological agents

**Figure 1:**
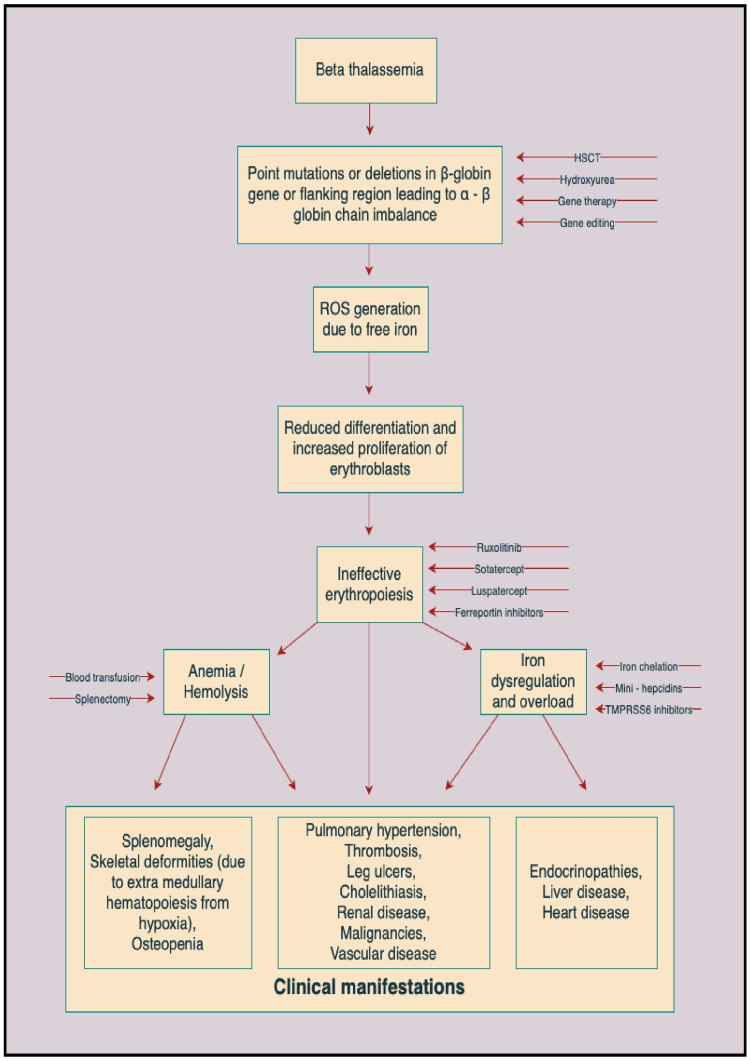
Mechanism of action of different treatment modalities in beta-thalassemia major HSCT: hematopoietic stem cell transplant, ROS: reactive oxygen species, TMPRSS6: transmembrane protease serine 6

Several treatment modalities like drugs that act by increasing hemoglobin (Hb) F (like hydroxyurea, azacytidine, thalidomide, and butyrates) or stimulate erythropoiesis (recombinant erythropoietin) have been tried in thalassemia with some success [3-6]. Among these drugs, thalidomide and hydroxyurea have been known to cause severe adverse effects, thus limiting their routine use in thalassemia treatment. Thalidomide has been associated with leucopenia, birth defects, neuropathy, and increased risk of thrombosis. Hydroxyurea is known to cause myelosuppression. Recently, newer treatment modalities targeting ineffective erythropoiesis are also being explored to treat beta-thalassemia (Figure 1). Drugs targeting ineffective erythropoiesis can be classified under the following groups, viz, activin II receptor traps (Luspatercept and Sotatercept), Janus-associated Kinase 2 (JAK2) inhibitors (Ruxolitinib), drugs targeting iron metabolism (mini hepcidins, serine protease TMPRSS6 inhibitors, ferroportin inhibitors, and apotransferrin F), macrophage manipulation therapy, and heat shock proteins [7].

Activin II receptor ligand traps

Mechanism of action: Growth and differentiation factor 11 (GDF11), an activin belonging to the transformation growth factor-β (TGF-β) family, plays a crucial role in erythropoiesis. GDF11 binds to activin type IIa receptor (ActRIIa) or type IIb receptors (ActRIIb) and induces the recruitment and phosphorylation of an activin type I receptor, which then phosphorylates the Smad2 and Smad3 intracellular signaling proteins. GDF11-ActRIIb-Smad2/3-dependent signaling is a key regulatory mechanism in proliferating erythroid precursors, as it controls their late-stage maturation. Growth differentiation factor 11 (GDF-11) has been postulated as the critical factor responsible for inhibiting late-stage erythroid differentiation and leading to ineffective erythropoiesis [8-9]. The activin II receptor ligand traps contain the extracellular domain of human activin receptor type IIb (ActRIIb), leading to reduced activin binding to the ActRIIb. Hence, they promote the terminal differentiation of erythroblasts with correction of maturation arrest. They reduce oxidative stress and the excess α-globin aggregates, which are responsible for the ineffective erythropoiesis [10-11]. Activin receptor-II binding traps include two agents, Sotatercept (ACE-011) and Luspatercept (ACE-536), which have shown promising results in thalassemia major.

Luspatercept

Luspatercept (ACE - 536) is a novel, soluble, recombinant fusion protein. Structurally, it has a modified extracellular domain of human activin receptor type IIb (ActRIIb) attached to the human IgG1 Fc domain [12-14].

Mechanism of action: Luspatercept enhances red blood cell (RBC) production by serving as a trap for ligands (belonging to the transforming growth factor-beta {TGF-β} superfamily) that inhibit late stages of erythropoiesis by diminishing Smad 2/3 intracellular signaling. Therefore, it promotes the differentiation and proliferation of late-stage erythroid precursors (normoblasts), improves hemoglobin concentration, and reduces the transfusion burden in thalassemia [12-16].

Pharmacokinetics: Luspatercept displays linear kinetics over doses of 0.2-1.25 mg/kg. A steady-state serum concentration is obtained after three subcutaneous doses (1 mg/kg or 1.25 mg/kg) administered every three weeks; a mean half-life (t_1/2_) of approximately 11 days is seen. The absorption of the drug is unaffected by the site of injection [12]. The pharmacokinetics of luspatercept are independent of age, gender, race, ethnicity, hepatic or renal impairment, RBC transfusion burden, baseline serum albumin or erythropoietin levels, beta-thalassemia genotype (β_0_/β_0_ vs. non - β_0_/β_0_), splenectomy, or the concomitant use of iron chelating agents [12-13].

Clinical trials: The BELIEVE study, a phase III trial (Clinical Trials.gov number NCT02604433), is a randomized, double-blind, placebo-controlled trial conducted on a total of 336 adult transfusion-dependent thalassemia (TDT) patients (receiving 6 to 20 units of packed red cells, with no transfusion-free period of >35 days, within 24 weeks before randomization). Participants were randomized to receive either luspatercept at a dose of 1 to 1.25 mg/kg or a placebo subcutaneously every three weeks for at least 48 weeks. The study's endpoints were an erythroid response, defined as the percentage of participants having a reduction in the transfusion burden of at least 33% from the baseline (with a reduction of at least 2 RBC units) over a 12-week interval (from weeks 13-24). Secondary endpoints included a reduction in the transfusion burden of at least 33% from baseline (with a reduction of at least 2 RBC units) during weeks 37-48. They reported that the patients with a reduced transfusion burden of at least 33% from baseline (with a reduction of at least 2 RBC units) during weeks 13-24 were significantly higher in the luspatercept group as compared to the placebo group (21.4% vs. 4.5%, p<0.001) along with a reduction in serum ferritin levels in the luspatercept group. Approximately 75% of patients had achieved at least a 33% reduction in transfusion burden during any 12-week period during the 48-week double-blind treatment with luspatercept [15-16]. 

Another open-label, dose-defining, non-randomized, uncontrolled, phase 2 study (Clinical Trials.gov number NCT01749540) was conducted in 31 non-transfusion dependent thalassemia (NTDT) patients (receiving < 4 units of RBCs within 8 weeks prior to day 1 cycle 1) and 32 TDT patients (requiring ≥ 4 units of RBCs every 8 weeks), with prior splenectomy or spleen size < 18 cm in the longest diameter by abdominal ultrasound. Luspatercept was administered subcutaneously every three weeks at a dose ranging from 0.2 to 1.25 mg/kg [17]. The erythroid response was defined as an increase in hemoglobin (Hb) of ≥ 1.5 g/dL from baseline for ≥ 2 weeks (in the absence of RBC transfusions) for NTDT and ≥ 20% reduction in RBC transfusion burden compared to pretreatment in TDT patients over a period of 12 weeks. A mean increase in Hb from a baseline of ≥ 1.5 g/ dL over two weeks was noted in 18 (58%) NTDT patients. In TDT patients, 26 (81%) achieved a transfusion burden reduction of ≥ 20% over 12 weeks at a dose of 0.6 to 1.25 mg/kg. The study recommended using luspatercept in a starting dose of 1 mg/kg (titration up to 1.25 mg/kg) [13].

A phase 2, double-blind, randomized, placebo-controlled, multicenter study (BEYOND trial, Clinical Trials.gov number NCT03342404) is underway to determine the efficacy and safety of Luspatercept as compared to placebo in adults with NTDT (defined as receiving 0 to 5 units of RBCs during the 24 weeks and RBC transfusion-free for at least ≥8 weeks prior to randomization). The study's primary endpoint is the estimation of the proportion of participants with an increase in Hb of ≥1.0 g/dl from baseline over a 12-week interval (from weeks 13-24) in the absence of RBC transfusions [18].

Dosing: Luspatercept is available commercially as a white lyophilized powder in single-dose vials (Reblozyl®, Acceleron Pharma - Celgene Corporation, 25 mg and 75 mg/vial) and is reconstituted with sterile water. The recommended dose for starting the therapy is 1 mg/kg subcutaneously once every three weeks. If there is no reduction in the patient’s transfusion requirement after at least two consecutive doses (6 weeks), the dose may be escalated to a maximum dose of 1.25 mg/kg. However, if still there is no response after nine weeks (3 doses) at a maximal dose or if the patient experiences unacceptable toxicity at any point of time, the drug must be discontinued [12]. No dose adjustment is needed in mild to severe hepatic insufficiency and mild to moderate renal disease [12].

Adverse events: In the BELIEVE trial, adverse effects (of all grades) were reported in >5% of patients treated with luspatercept. The commonly reported side effects were back pain (27%), upper respiratory tract infection (26%), headache (26%), bone pain (20%), arthralgia (19%), pyrexia (16%), cough (14%), fatigue (13%), oropharyngeal pain (13%), diarrhea (12%), dizziness (11%), myalgia (9%), nausea (9%), abdominal pain (8%), hypertension (8%), and hyperuricemia (7%) [13]. Serious adverse effects (3.6%) reported were cerebrovascular accidents and deep vein thrombosis (1%). The patients must also be cautioned about the potential for immunogenicity, whereby the presence of neutralizing antibodies against luspatercept can decrease its serum concentration [12,15-16].

Safety: Currently, there are no absolute contraindications for luspatercept. However, caution must be exercised while prescribing luspatercept to patients with thromboembolic events, hypertension, pregnancy, lactation, and females of reproductive potential [12]. Animal studies have reported an increased risk of developing hematologic malignancies at doses of 10 mg/kg, a dose approximately eight-fold higher than the maximum recommended human dose (MRHD). Fertility studies conducted in rats had reported effects on female (but not male) fertility at doses almost seven times higher than the MRHD. Female patients with beta-thalassemia being considered for Luspatercept treatment should undergo pregnancy testing before starting treatment and should also be advised to use contraception during treatment and three months after receiving the final dose. Lactating females should not breastfeed while undergoing treatment and even three months after receiving the last dose [12]. Currently, the safety and efficacy of luspatercept in children with thalassemia have not been established [12].

Regulatory consideration: Luspatercept was approved by the United States Food and Drugs Administration (US FDA) on November 8, 2019, for use in TDT patients [19-20].

Sotatercept

Sotatercept (ACE-011) is a dimeric recombinant fusion protein that consists of an extracellular domain of the human activin type IIa receptor (ActRIIa) linked to the Fc portion of human immunoglobulin G1 (IgG1) [21-23].

Mechanism of action: Sotatercept acts as a ligand trap and binds activin A and other proteins of the TGF-β superfamily that act as negative regulators of late-stage erythropoiesis, preventing their downstream Smad 2/3 signaling and thereby acts to increase mature RBCs [9,22-24].

Pharmacokinetics: Sotatercept in a dose of 0.1 to 1 mg/kg given subcutaneously follows first-order linear and time-independent pharmacokinetics with an elimination half-life of 21 to 23 days [25].

Clinical trials: A phase 1 study was conducted in a group of 31 healthy volunteers (post-menopausal women) to evaluate the safety, pharmacokinetic, and pharmacodynamic properties of Sotatercept [25]. The drug was administered subcutaneously at a dose of 0.1, 0.3, or 1 mg/kg every 28 days. A dose-dependent increase was observed in the level of hemoglobin (Hb), hematocrit, and RBC counts; the results were clinically significant and sustained for up to four months.

Another phase 2, open-label, dose-defining, multicenter study attempted to determine the safety and tolerability of Sotatercept in 16 TDT and 30 NTDT adult patients with beta-thalassemia [26]. Transfusion dependence was defined as receiving ≥2 RBC units every 30 days for ≥168 days before study enrollment, with no transfusion-free period of >45 consecutive days during this period. Non-transfusion dependence was defined as ≤1 transfusion during the 168 days before study enrollment. Sotatercept was given subcutaneously at doses of 0.1, 0.3, 0.5, 0.75, or 1 mg/kg every three weeks for ≤22 months, with the median duration of treatment being 13.8 months for TDT patients and 19.6 months for NTDT patients. The study's endpoints were a ≥20% reduction in transfusion burden for 24 weeks in TDT patients and an increase in hemoglobin level of ≥1.0 g/dL for 12 weeks in NTDT patients. Of the 16 TDT patients who received Sotatercept, 10 (63%) achieved a transfusion burden reduction of ≥20% lasting for ≥24 weeks; seven (44%) achieved a reduction of ≥33%, and two (13%), a reduction of ≥50%. The mean change in the Hb level from baseline to the end of treatment was 0.7 g/dL. In 30 NTDT patients, 18 (60%) and 11 (37%) patients achieved a mean Hb increase of ≥1.0 g/dL and ≥1.5 g/dL, respectively, sustained for ≥12 weeks. Overall, Sotatercept had a good safety profile, having been tolerated well by most patients.

Dosing: At present, no recommendations exist regarding the optimum dose of Sotatercept. Based on the phase 2 study, a dose of ≥0.3 mg/kg and ≥0.5 mg/kg given subcutaneously every three weeks is recommended in NTDT and TDT, respectively [22].

Adverse effects: Bone pain, arthralgia, back pain, asthenia/fatigue, hypertension, headache, and cough were commonly reported side effects. Injection site erythema, pyrexia, extramedullary hematopoiesis, ventricular extrasystoles, and hypersensitivity were rarely reported. Discontinuation of treatment due to grade 3 or 4 adverse events was not reported. No significant changes in laboratory values for liver and kidney function were noted [22,24].

Regulatory approval: Sotatercept has not yet been licensed for use in beta-thalassemia. More studies are needed to define its position in the treatment algorithm for beta-thalassemia patients. Presently, the drug is not available in the market.

Janus-associated kinase (JAK) inhibitors

Although the more powerful JAK inhibitors act by aggravating anemia, weak JAK inhibitors may decrease ineffective erythropoiesis. Weak JAK2 inhibitors act by the anti-inflammatory mechanism and induce apoptosis of the more pathological erythroid precursors, thus inducing more efficient erythropoiesis. Past studies on NTDT and TDT animal models revealed that JAK2 inhibition improved ineffective erythropoiesis and reversed splenomegaly, thereby paving a path for JAK1 and JAK2 inhibitors [27-28].

Ruxolitinib

It is an oral JAK inhibitor that binds and inhibits tyrosine kinases JAK 1 and 2. It is currently approved for polycythemia vera and myelofibrosis, although promising results are emerging in beta-thalassemia [29-30].

Mechanism of action: Ruxolitinib acts on the initial stages of erythropoiesis. Erythropoietin (EPO) binds to its cell surface receptor, activating the cytoplasmic JAK2, which in turn activates downstream pathways involved in erythropoiesis, including the signal transducer and activator of transcription 5 (STAT5), which results in increased proliferation, differentiation, and survival of erythroid precursors [31-32]. Elevated EPO production is seen in animals affected with beta-thalassemia. This pathway is constantly active, leading to extramedullary erythropoiesis and stress erythropoiesis in the spleen [27-28,31].

Pharmacokinetics: Ruxolitinib has an oral bioavailability of 95%, which is not affected by concomitant intake of food, with 97% of the drug bound to plasma proteins. The peak plasma concentration is achieved one to two hours after administration. It is metabolized primarily by CYP3A4 and to a lesser extent by CYP2C9 and is eliminated by the kidneys. The half-life ranges from three to six hours [33-34].

Clinical trials: A single-arm, multicenter, phase 2 study (TRUTH study) over a span of 30 weeks evaluated the efficacy and safety of ruxolitinib in 30 adult patients with TDT (on regular transfusion regimen requiring ≥2 RBC units within 4-week intervals for 24 weeks before enrollment) and splenomegaly (≥450 cm^3^) [35]. Ruxolitinib was administered orally starting with a dose of 10 mg twice daily (5/10 mg increments with the maximum dose being 25 mg) for 30 weeks. A reduction in mean spleen volume of 19.7% and 26.8% from baseline at week 12 and week 30, respectively, and about a 6% reduction in transfused RBC volume was observed. However, no significant improvement in pre-transfusion hemoglobin levels or reduction in the transfusion requirement was seen. An increase in hepcidin levels was seen, although associated significant changes in iron parameters, such as serum iron and ferritin, were not seen. Due to the reasons mentioned above, the study could not proceed to phase 3 [35-36].

Dosing: Currently, there are no dosage recommendations for ruxolitinib in thalassemia.

Adverse effects: The commonly reported side effects were upper respiratory tract infection (27%), nausea (20%), upper abdominal pain (17%), anemia (17%), diarrhea (17%), and weight gain (17%). Serious adverse events reported were anemia, nausea, vomiting, pyrexia, drug-induced liver injury, and pneumonia [35].

Regulatory approval: Ruxolitinib is not approved yet for use in beta-thalassemia.

Iron metabolism modulation

Hepcidin is the key molecule regulating iron metabolism. There is downregulation of hepcidin in thalassemia due to ineffective erythropoiesis, which potentiates iron overload [7]. Thus, novel therapeutic modalities have been explored to alter iron metabolism and improve erythropoiesis in thalassemia.

Mini Hepcidins

Iron absorption can be reduced by using mini hepcidins. Casu et al. conducted studies in mice models resembling human NTDT and TDT phenotypes and reported an improvement in ineffective erythropoiesis, anemia, a reduction in iron overload, and a decrease in splenomegaly [37-38]. However, two phase 2 trials aimed at assessing the safety and efficacy of mini hepcidins were prematurely terminated due to efficacy issues [39-40].

TMPRSS6 and Erythroferrone

TMPRSS6, a serine protease that suppresses hepcidin expression, has also emerged as a promising approach. In a study by Nai et al. in Hbb^th3/+^ mice, TMPRSS6 gene deletion was found to improve anemia, ineffective erythropoiesis, and splenomegaly [41]. A phase 2, multicenter, open-label study by IONIS Pharmaceuticals (Carlsbad, California) is now underway to study the effects of IONIS TMPRSS6-LRx in adult NTDT patients (≥18 years of age). A single injection of IONIS TMPRSS6-LRx at multiple dose levels will be administered to participants subcutaneously every four weeks for 24 months. The study will measure the increase in hemoglobin from baseline and decrease in liver iron concentration from the baseline in the study participants [42].

In mouse models of beta-thalassemia intermedia, Kautz et al. found that erythroferrone ablation could restore normal levels of hepcidin and suggested that erythroferrone could result in reduced hepcidin production in beta-thalassemia [43]. Smesam et al. conducted a study on 60 TDT patients and compared them with 30 healthy controls [44]. They reported a significant correlation between the reduction in hepcidin levels and an increase in erythroferrone, although no direct correlation of erythroferrone was found with the other measured iron status parameters.

Ferroportin Inhibitors

A novel agent, VIT-2763, which is a small oral molecule, acts as a ferroportin inhibitor. It was found to block iron efflux by competing with hepcidin for ferroportin binding and triggering ferroportin internalization and ubiquitination. In Hbb^th3/+^ mice models, VIT-2763 improved anemia, erythropoiesis, reduced iron overload in the liver, and reduced myelopoiesis in the spleen [45].

A phase 1 double-blind, randomized, placebo-controlled, dose-escalation study was conducted in healthy adult male and female volunteers to determine the safety, tolerability, pharmacokinetic properties, and pharmacodynamic effects of VIT-2763. A temporary decrease in mean serum iron levels and mean transferrin saturation was seen, along with a shift in mean serum hepcidin peaks, after administrating VIT-2763 [46]. A randomized, double-blind, placebo-controlled parallel-group trial is currently underway to determine the safety, tolerability, and efficacy of multiple doses of VIT-2763 versus placebo in NTDT patients [47].

Apotransferrin

The administration of apotransferrin (apo-TF) in mice with thalassemic phenotype revealed that apo-TF administration improved ineffective erythropoiesis by increasing the proportion of mature erythroid precursors [48]. Human trials have not been conducted to explore the use of apotransferrin.

Macrophage manipulation therapy

Although macrophages’ interaction with erythrocytes is critical for erythropoiesis, they have also been found to promote stress erythropoiesis, thus limiting sustained erythropoiesis in thalassemia. Ramos et al. used clodronate-loaded liposomes to deplete macrophages in a mouse model of thalassemia [49]. They found that the treatment rapidly ameliorated the anemia and improved erythropoiesis, with a concurrent reduction in spleen size, improved iron status, and a decrease in the α-globin chain. This mechanism can also be explored with human trials in the future.

Heat shock proteins (HSP)

HSP 70, a molecular chaperone, normally protects GATA-1 (a fundamental erythroid transcription factor) from Caspase-3 mediated cleavage during normal terminal erythroid differentiation. This occurs by nuclear accumulation of HSP 70 during terminal maturation [7]. However, HSP70 is sequestrated in the cytoplasm by excess free alpha-globin chains in beta-thalassemia, thereby preventing its nuclear localization and ultimately leading to ineffective erythropoiesis [50]. Therefore, decreasing HSP70 nuclear export (export being usually mediated by exportin 1, i.e., XPO1) could be a potential therapeutic approach. This was demonstrated by Guillem et al. by using an XPO1 inhibitor to increase the amount of nuclear HSP70, thus rescuing GATA-1 expression and improving terminal differentiation to ameliorate ineffective erythropoiesis in β-thalassemia [51].

Table 1 provides a snapshot of ongoing/completed clinical trials on the newer pharmacological drugs aimed at improving the ineffective erythropoiesis.

**Table 1:** Clinical trials for drugs affecting ineffective erythropoiesis in beta-thalassemia patients

Drug	Clinical Trial Identifier	Phase of Clinical Trial	Status	Location	Number of participants	Conclusion
Luspatercept [12,17]	NCT01749540	II	Completed	Italy	64	Increased hemoglobin concentration, reduced transfusion burden, and reduced liver iron content
Luspatercept [52]	NCT02268409	II	Completed	Italy, Greece	51	Increased hemoglobin concentration and reduced transfusion burden
Luspatercept, Placebo-controlled [18]	NCT03342404 (BEYOND Trial)	II	Active, not recruiting	Multinational	145	-
Luspatercept [53]	NCT04143724	II	Recruiting	Multinational	48	-
Luspatercept, Placebo-controlled [16]	NCT02604433 (BELIEVE Trial)	III	Active, not recruiting	Multinational	336	Reduced transfusion burden
Luspatercept [54]	NCT04064060	III	Recruiting	Multinational	665	-
Sotatercept [26]	NCT01571635	II	Active, not recruiting	Multinational	46	-
Ruxolitinib [36]	NCT02049450 (TRUTH Trial)	III	Completed	Multinational	30	No change in transfusion requirement, reduction in spleen volume from baseline observed

Economic considerations: Currently, only Luspatercept (FDA approved) and ruxolitinib (non-FDA approved) are available in the market. Approximately, the price of a vial of 25 mg Luspatercept costs $3700, and a vial of 75 mg Luspatercept costs $11,000 [55]. Meanwhile, a supply of 60 ruxolitinib tablets of 10 mg costs nearly $15,000 [56]. The high costs are a limitation in low and middle-income countries, although if generic versions are made available, the cost can be reduced. This would benefit countries with resource limitations, adding to the spectrum of treatments available for beta-thalassemia.

## Conclusions

Therapeutic management of beta-thalassemia remains a big challenge, especially in low and middle-income countries due to resource limitations. Available treatment options are mainly supportive therapy in the form of regular transfusions and iron chelation. Thus, drugs addressing ineffective erythropoiesis may have a role as adjunctive therapy but at present, recommending the routine use of these novel agents in thalassemia warrants investigations in larger phase 3 trials.
